# Sociodemographic Factors Associated with Adherence to Dietary Guidelines in Women with Gestational Diabetes: A Cohort Study

**DOI:** 10.3390/nu13061884

**Published:** 2021-05-31

**Authors:** Sara Mustafa, Jane Harding, Clare Wall, Caroline Crowther

**Affiliations:** 1 Liggins Institute, The University of Auckland, Auckland 1023, New Zealand; sara.mustafa@auckland.ac.nz (S.M.); j.harding@auckland.ac.nz (J.H.); 2 Faculty of Medical and Health Sciences, The University of Auckland, Auckland 1023, New Zealand; c.wall@auckland.ac.nz

**Keywords:** pregnancy, diet therapy, patient compliance, clinical practice guidelines

## Abstract

Dietary advice is the cornerstone of care for women with gestational diabetes mellitus (GDM). However, adherence to this advice is variable. We aimed to identify the proportion of women with GDM who adhere to the New Zealand nutrition guideline recommendations and assess the sociodemographic factors linked to dietary adherence. We assessed dietary intake at 36 weeks’ gestation in a cohort of 313 women with GDM and compared this with the dietary recommendations for the management of GDM. Associations between maternal characteristics and dietary adherence were assessed using ANOVA, chi square, logistic regression, and linear regression tests. Women with GDM had an average adherence score of 6.17 out of 10 to dietary recommendations, but no one adhered to all the recommendations. Adherence to recommendations was lowest for saturated fat, and wholegrain breads and cereals. While 85% visited a dietitian, only 28% of women achieved their recommended weight gain. Maternal factors associated with lower dietary adherence were primiparity, no previous history of GDM, being underweight, and smoking. Adherence to the dietary recommendations by women with GDM in New Zealand for the management could be improved. Further research is needed to identify ways for women with GDM to improve their dietary adherence.

## 1. Introduction

Gestational diabetes mellitus (GDM) is glucose intolerance first recognised in pregnancy and is managed by providing dietary and lifestyle advice, together with pharmacological support, such as oral hypoglycaemics and/or insulin when needed [1]. Poorly controlled maternal glucose concentrations increase the risk of complications for the woman and her infant during and after the pregnancy [2]. Dietary therapy alone has been reported to be effective in controlling maternal blood glucose concentrations in 70% of women [3], thus reducing the need for pharmacological treatments, such as insulin or oral hypoglycaemics [4]. As dietary advice is recognised worldwide as the first line treatment in the management of GDM, assessment of dietary adherence in women with GDM and factors that may influence this is key when evaluating care practices [5]. 

Adherence is defined by the World Health Organisation as ‘the extent to which a person’s behaviour—taking medication, following a diet, and/or executing lifestyle changes, corresponds with the agreed recommendations from a health care provider’ [6]. Lack of adherence of a patient to clinical recommendations, whether it be for prevention or treatment, can reduce the effect of the desired outcome [7]. Adequate adherence to dietary interventions is likely to reduce the risk of developing complications of GDM, and so avoidance of treatments that require further adherence [8]. Recognising factors that influence dietary adherence by women with GDM to clinical practice guideline recommendations may help identify ways to improve and maintain adherence in high-risk populations [9].

Adherence of women with GDM to the New Zealand clinical practice guidelines dietary recommendations has not been previously investigated. This study aimed to assess the proportion of women with GDM who adhere to the dietary recommendations in New Zealand and the sociodemographic factors associated with adherence. 

## 2. Materials and Methods

### 2.1. Study Population

This was a cohort study nested within the TARGET Trial. The TARGET Trial (Australian New Zealand Trial Registry: ACTRN12615000282583) is a stepped-wedge cluster randomised trial in women diagnosed with GDM that assessed the effect of less tight compared with tighter glycaemic treatment targets on maternal and infant health [10].

Women with a singleton pregnancy were eligible for this cohort study if they participated in the TARGET Trial, completed a food frequency questionnaire (FFQ) [11] in late pregnancy (close to 36 weeks’ gestation), and data were available for at least one of the following maternal and sociodemographic characteristics: maternal age, maternal history of GDM, family history of diabetes, parity, body mass index (BMI), ethnicity, smoking status, gestational age at trial entry and New Zealand Deprivation Index (NZDep 2013) [12]. Data were collected for the TARGET Trial [10] and included maternal dietary intake using a validated semi-quantitative FFQ late in pregnancy (36 weeks’ gestation) [11] and maternal and sociodemographic information.

### 2.2. Dietary Adherence

Dietary adherence was assessed by comparing the consumed food reported in the FFQ in late pregnancy with the New Zealand dietary recommendations from the 2014 New Zealand guideline Screening, Diagnosis and Management of Gestational Diabetes in New Zealand [13] and the 2006 New Zealand guideline for Healthy Pregnant and Breastfeeding Women [14]. These guidelines are those most widely used for the dietary management of GDM in New Zealand [15]. Both were commissioned by the New Zealand Ministry of Health and complement each other, with the 2014 guideline providing specific dietary recommendations for women with GDM [13,14]. 

The recommendations from these two guidelines were categorised into food-related recommendations and non-food-related recommendations. The food-related recommendations included daily intake of energy, carbohydrate, saturated fat, vegetables and fruit, breads and cereals (and wholegrain), milk and milk products (and reduced-fat), and lean meat, poultry, seafood, eggs, nuts and seeds, and legumes. The non-food-related recommendations included ‘visited a dietitian’ and ‘achieved recommended weight gain’. The dietary recommendation to ‘consume lean protein’ made in the 2014 guideline was assessed by intake of the food group ‘lean meat, poultry, seafood, eggs, nuts and seeds, and legumes’ [13]. To ensure no repetition of scores for that category, the food group recommendation was used to assess adherence instead of the recommendation ‘consume lean protein’, as this included greater detail on serving size. 

As no scoring system for dietary adherence has been validated for New Zealand guidelines specific to women with GDM, a scoring tool was developed to assess dietary adherence to food-related guidelines (Table 1). A score between zero and one was calculated for each recommendation as the ratio of reported dietary intake to the recommended intake, where a maximum score of one was assigned for full adherence and a score of zero for no adherence to the recommendations. A participant’s individual dietary scores were then summed to assess overall adherence to the food-related guideline recommendations on a continuous scale, with a score of 10 indicating full adherence to all food-related recommendations, while a score of zero indicated no adherence. 

The non-food-related dietary recommendations were the use of dietary therapy as first-line therapy, distribution of meals and gestational weight gain. A recent survey on dietetic management of GDM conducted among dietitians in New Zealand showed 86% of dietitians reported using the 2006 guideline and 79% the 2014 guideline [15]. In this study, we have assumed that during a visit to the dietitian, advice consistent with these guideline recommendations on meal and snack consumption, distribution of carbohydrates, and consideration of patient preferences and culture would have been given to the women [15]. Therefore, if the women had visited a dietitian after the diagnosis of GDM, they were considered to have adhered to those recommendations. If maternal weight gain during pregnancy fell within the recommendation for the BMI’s range, the woman was considered to have adhered to the recommended weight gain. 

### 2.3. Statistical Analysis

Statistical analysis was conducted using IBM SPSS Statistics 26 statistical software (version 26, IBM Corp, Armonk, NY, USA). Baseline characteristics of eligible women were summarised using descriptive statistics. Outliers were removed if the maternal energy intake was reported as greater or less than 1.5 times the interquartile range. 

The associations between adherence scores for the food-related recommendations and maternal characteristics and demographics were explored using ANOVA and linear regression with Fisher’s least significant difference (LSD) post-hoc analysis where appropriate. The associations between non-food-related recommendations and maternal characteristics and demographics were assessed using χ^2^ test and logistic regression. A two-sided *p*-value of <0.05 was considered statistically significant.

## 3. Results

### 3.1. Study Population

A total of 313 women with GDM were eligible for and included in this cohort study. Most were of European ethnicity, followed by Asian, Pacific, and Māori (Table 2). Nearly a third of women were living in the most deprived quintile areas, and over half were obese. The median age was 33.0 (interquartile range: 29.0–36.0) years and 44% were in their first pregnancy. Approximately one in five women had been diagnosed with GDM in a previous pregnancy, and over half of the women had a family history of diabetes. 

### 3.2. Dietary Adherence Scores

The mean dietary adherence score was 6.17/10 (standard deviation: 1.22, range: 2.35–9.49). No one adhered to all ten food-related recommendations. The proportion of women who adhered to the recommended energy intake was 80.8% (*n* = 253) and for carbohydrate intake was 84.3% (*n* = 264). No one adhered to the saturated fat intake recommendation, with the majority of woman receiving a score of zero (*n* = 262, 83.7%) (Table 3). 

Adherence to recommendations for the five main food groups (vegetables, fruits, breads and cereals, milk and milk products, and lean meat, meat alternatives and eggs) was met by only 4.5% (*n* = 14) of the women. Adherence to those recommendations for the main five food groups plus the additional two healthier alternatives (wholegrain breads and cereals, and reduced-fat milk and milk products) was met by only one percent (*n* = 3) of women. Adherence was greatest for the vegetable food group, with nearly half of the women (*n* = 152, 48.6%) consuming the recommended serving (Table 3). This was closely followed by adherence to the recommended intakes for fruit (*n* = 142, 45.4%), milk and milk products (*n* = 121, 38.7%) and lean meat, meat alternatives and eggs (*n* = 107, 34.2%). Adherence to the recommended intake was lowest for the breads and cereal food group (*n* = 39, 12.5%). There was also poor adherence to the healthier alternatives in the milk and milk products food group, and the breads and cereal food group. Only 7% (*n* = 20) adhered to the advised intake of reduced-fat milk and milk products, and only 3% of women (*n* = 9) consumed the recommended servings of wholegrain breads and cereals (Table 3).

Adherence to both non-food-related recommendations (visitation to a dietitian and recommended gestational weight gain) was met by almost 25% of women with GDM (*n* = 78, 24.9%) (Table 3). A higher proportion of woman visited a dietitian (*n* = 269, 85.9%) than achieved their recommended gestational weight gain (*n* = 88, 28.1%) (Table 3). Of the 225 (71.9%) that did not achieve the recommended gestational weight gain, 110 (48.9%) women gained weight below the recommended range while 115 (51.1%) women gained weight above the recommended range. Nearly all the women (*n* = 110, 95.7%) who gained weight above the recommended range were overweight or obese.

### 3.3. Adherence to Food-Related Recommendations and Maternal Sociodemographics 

Maternal age, parity, gestational age at trial entry, socioeconomic deprivation, family history of diabetes, smoking at trial entry and visitation to a dietitian were not associated with total dietary adherence score, and adherence to the recommended intake of energy, carbohydrate, and saturated fat (Table 4).

Maternal ethnicity was associated with adherence to the recommended energy intake, but not to total dietary adherence score, carbohydrate, or saturated fat intake (Table 4). Women from other ethnicities had significantly lower adherence scores for the recommended energy intake compared to Pacific, Asian and European women, although not compared to Māori women. 

Women of normal weight had greater adherence to the energy and carbohydrate recommendations compared to women who were underweight or overweight, but not those who were obese (Table 4). However, women who were underweight had greater adherence to the saturated fat intake recommendation compared to women who were normal, overweight, or obese.

A previous history of GDM was associated with higher dietary adherence scores for energy and carbohydrate but lower scores for saturated fat (Table 4). Women who achieved the recommended weight gain recommendations had higher dietary adherence scores for saturated fat compared to women who had gained weight higher or lower to the gestational weight gain recommendation.

Dietary adherence to the recommendations differed according to maternal sociodemographic factors (Table 5).

Pacific women had lower adherence to the recommended intake of milk and milk products compared to Māori, Asian and European women; however, they had higher adherence to the recommended bread and cereals intake (Table 5).

Women in the least deprived areas (NZDep 1 to 2) had higher adherence to the recommended intake of milk and milk products compared to women living in the most deprived areas (NZDep 7 to 10) and greater adherence to the recommended intake of reduced-fat milk and milk products compared to slightly more deprived areas (NZDep 3 to 4) (Table 5). 

Women over 40 years of age had greater adherence to the recommended intake of wholegrains cereals and breads compared women between the age of 25 to 39 years, but no difference was found between women of 20 to 24 years of age (Table 5). Multiparous women and women with a previous pregnancy with GDM had higher dietary adherence scores to the recommended intakes of bread and cereal food group, as well as of wholegrain bread and cereal group, compared to primiparous women and women who had not had GDM before.

Women who smoked had lower adherence to the recommendations for wholegrain bread and cereals compared to women who did not smoke (Table 5). Women who visited a dietitian at least once had greater adherence to the recommended intake of breads and cereals than women who did not visit a dietitian after a GDM diagnosis. Maternal BMI, gestational age at trial entry and a family history of diabetes were not associated with adherence to any of the recommendations about food groups.

### 3.4. Adherence to Non-Food-Related Recommendations and Maternal Characteristics

No associations were found between a dietitian visit and maternal ethnicity, age, BMI, parity, previous history of GDM, socioeconomic deprivation, family history of diabetes, or smoking status. (Table 6). Women were less likely to visit a dietitian after a diagnosis of GDM as gestational age at trial entry increased (OR = 0.806, *p* = 0.011, Table 6).

No associations were found between achieving gestational weight gain and maternal age, BMI, parity, gestational age at study entry, previous history of GDM, socioeconomic deprivation, family history of diabetes, or smoking status (Table 6). The proportion of Māori women (*n* = 2, 6.5%) who achieved the recommended gestational weight gain was lower than for other ethnicities (Table 6). 

## 4. Discussion

### 4.1. Adherence to Dietary Recommendations 

Overall, adherence to the dietary recommendations by women with GDM in New Zealand for the management was moderate, with an average adherence score of 6.17/10. However, no women adhered to all the dietary recommendations. Approximately four out of five women adhered to the energy and carbohydrate recommendations, but a similar proportion of women did not adhere to the saturated fat intake recommendation. This suggests that most women with GDM in New Zealand consumed an adequate number of calories and carbohydrates but consumed greater than the recommended intake of saturated fat. 

Fewer than 4.5% of women adhered to recommendations for all the five main food groups in this study. This is slightly higher than the 3% of pregnant women with and without GDM who previously were found to have adhered to dietary recommendations in New Zealand [16], the 3.5% of women in Ontario, Canada [17] and the 0% in one report from New South Wales, Australia [18]. The dietary adherence rates to the individual food group recommendations in this study ranged from 2.9% to 48.6%. While fewer than half of women adhered to the recommendations for any one of the food groups, the median dietary adherence scores indicate that most women were close to adhering to the recommended food servings in some of the food groups. For example, the scores for intake of vegetables (score = 0.98) and fruits (score = 0.93) showed that the median intake was 3.92 servings instead of the recommended four servings of vegetables, and 1.86 servings instead of the recommended two servings of fruits. 

Globally, pregnant women adhere poorly to the recommended intake for the vegetables food group, and the breads and cereals food group [19]. However, from the five food groups, women in this study adhered poorly to the recommended breads and cereals intake. The best adherence was to the recommended intake of vegetables. The dietary management of GDM emphasises appropriate carbohydrate intake from low glycaemic food sources, which may explain why women in this study had low consumption of breads and cereals and high vegetable intake [15]. 

Higher adherence rates were previously reported among pregnant women with and without GDM in New Zealand, compared with women in this study for the recommended intake of fruits (82% vs. 45.4%), bread and cereals (26% vs. 12.5%) and reduced-fat milk and milk products (10% vs. 6.4%) [16]. However, women in this study had higher adherence to the recommended intake for the vegetables (27% vs. 48.6%), milk and milk products (28% vs. 38.7%) and lean meat, meat alternatives or eggs (21% vs. 34.2%) food groups. Compared to the findings in this study, lower adherence to the recommendations across all five food groups was found in a study of pregnant women in Australia, which reported that no woman adhered to all the five food group recommendations [18]. 

Adherence to the non-food recommendations varied greatly. Over 85% of women visited a dietitian after GDM diagnosis. These results are comparable to findings in Switzerland [20], and a survey conducted amongst dietitians in New Zealand [15]. However, just under 30% of women with GDM achieved their recommended weight gain during pregnancy in this study. Adherence to the recommended gestational weight gain among women with GDM was found among 38.6% of women in Beijing, China [21] and 37.9% of Polish women [22]. Furthermore, among obese Portuguese women with GDM, 35.1% adhered to the recommended weight gain during pregnancy, which is greater than the 25.4% of obese women who adhered to this recommendation in this study [23]. 

### 4.2. Dietary Adherence and Maternal Sociodemographic Characteristics 

Most maternal sociodemographic factors were associated with adherence to the dietary recommendations for the management of GDM in New Zealand. From all the maternal sociodemographic factors explored, a previous pregnancy with GDM was one of the main factors that was associated with better dietary adherence to the clinical practice guidelines in this study. A previous history of GDM was associated with a higher total dietary adherence score and with greater adherence to five of the ten dietary recommendations: energy, carbohydrate, saturated fat, the bread and cereal food group, and the wholegrain bread and cereal food group. Knowledge about maternal dietary intake that may negatively impact the baby’s health was reported to enhance behaviour change in pregnant women in New Zealand [24]. For women with a previous pregnancy with GDM, this may have led to improved adherence to most of the recommendations due to prior knowledge about diabetic management compared to women receiving dietary advice for GDM for the first time. 

Ethnicity was associated with achieving the recommended gestational weight gain, which is consistent with reports in the literature [25,26,27]. Nine out of ten pregnant Māori women with GDM did not achieve the recommended weight gain, which is similar to the reported proportions in previous findings [25]. Furthermore, Pacific women had the greatest adherence to the recommended intake of breads and cereals food group, which is comparable to a previous report [16]. Pacific and Asian women had the lowest intake of milk and milk products among ethnicities in New Zealand, which has also been consistently found in the literature [16].

A first pregnancy was found to be associated with less adherence to the recommended intake of breads and cereals food group, which was also reported among pregnant women in Ontario, Canada [17]. In contrast to previous studies in the New Zealand pregnant population, socioeconomic status was associated with dietary adherence to the recommended intake of milk and milk products among women with GDM [16], with women in the least deprived areas having better adherence to the milk and milk products group recommendations. Individuals living in higher socioeconomic deprivation areas tend to have less food security and access to food [28]. Furthermore, food insecurity is heightened in females compared to males, and in individuals between the ages of 25 and 44 years of age, which is characterised by over 95% of the women in this study [28]. The reduced consumption of milk and milk products and other food staples in New Zealand is recognized as a primary concern among those that are food insecure [29]. 

### 4.3. Improving Dietary Adherence 

Targeting modifiable factors may improve adherence to the dietary recommendations for the management of GDM. In this study, being underweight and smoking were associated with poorer dietary adherence. However in a Danish study, pregnant women with GDM with a lower BMI (median BMI: 28) were more likely to adhere to the dietary guidelines than women with a higher BMI (median BMI: 30) [30]. 

Even though pregnancy is thought to be one of the most receptive times in adulthood to modify behaviour, the physiological changes that arise with pregnancy can influence behaviour change [31]. A qualitative study that included women with GDM described that morning sickness in the latter half of pregnancy, lack of education and food aversions affected adherence to the recommended gestational weight gain [32]. Furthermore, meals cooked by family members according to the GDM diet recommendations encouraged women to increase vegetable intake [33].

The importance of visiting a dietitian after a GDM diagnosis is highlighted in this study, as women who visited a dietitian adhered better to the recommendations for breads and cereals food group than those who did not visit a dietitian. Inconvenient appointment timing, limited access to health professionals and lack of access to transportation to attend a session are reported to be some reasons women do not visit a dietitian after being diagnosed with GDM [34,35]. 

### 4.4. Study Strengths and Limitations 

While the findings of this study are important to identify how well pregnant women adhere to the recommended guidelines for the management of GDM, the study did have some limitations. Dietary adherence was assessed using an FFQ which can over- or underestimate dietary intake and relies greatly on participant memory. However, the FFQ used in this study was validated, developed for the New Zealand population and is suitable for a population-based sample [11]. Furthermore, the development of an index to assess adherence, diet quality and food diversity may be useful to provide an overall assessment of dietary intake among the population [36]. The scoring tool was not validated to assess dietary adherence to the food-related dietary recommendations. However, dietary adherence was assessed in this study by calculating dietary intake as ratios of the recommended serving sizes to provide a more graduated estimate of maternal intake similar to previous cohort studies [37,38]. As the guidelines had no established maximum cut-off of servings for the food recommendations, women who ate more than the recommended minimum servings would have scored well on the adherence assessment. Therefore, it is unknown whether maternal factors were associated with intake greater than the minimum recommended amount. Our study did not have access to maternal educational data but did report on the socioeconomic deprivation index (NZDep) that is strongly correlated to the education level achieved.

This study highlights the need to explore ways to improve adherence to the dietary recommendations among women with GDM in New Zealand. Additional research should examine the barriers and enablers for healthier eating among women with GDM. Interventional studies should identify optimal strategies to help women with GDM improve their adherence to dietary recommendations.

## 5. Conclusions 

In New Zealand women with GDM, adherence to all clinical guideline dietary recommendations can be improved. Adherence is also related to a range of maternal sociodemographic factors. Future research should focus on identifying ways to improve dietary adherence, particularly in higher risk groups.

## Figures and Tables

**Table 1:** Food-related recommendations from New Zealand dietary clinical guidelines, their cut-off values and dietary adherence score [13,14].

Food-Related Recommendations	Intake per Day	Dietary Scores
Energy	≥1800 kcal	Maximum score of 1 if intake is ≥1800 kcal/day. If intake is <1800 kcal/day, score is calculated as:
		Caloric intake in kcal/1800
Carbohydrate	≥175 g	Maximum score of 1 if intake is ≥175 g/day. If intake is <175 g/day, score is calculated as:
		Carbohydrate intake in grams/175
Saturated fat	<10% of energy intake	Maximum score of 1 if intake is <10% of energy intake. If intake is ≥ 10%, score is calculated as:
		([10 − % of saturated fat intake]/10) + 1 Minimum score of 0 can be achieved.
Vegetables	≥four servings	Maximum score of 1 if intake is ≥four servings per day. If intake is <four servings, score is calculated as:
		Servings per day/4
Fruits	≥two servings	Maximum score of 1 if intake is ≥ two servings per day. If intake is <two servings, score is calculated as: Servings per day/2
Breads and cereals	≥six servings	Maximum score of 1 if intake is ≥six servings per day. If intake is <six servings, score is calculated as:
		Servings per day/6
Wholegrain breads and cereals	≥six servings	Maximum score of 1 if intake is ≥six servings per day. If intake is <six servings, score is calculated as:
		Servings per day/6
Milk and milk products and alternatives	≥three servings	Maximum score of 1 if intake is ≥three servings per day. If intake is <three servings, score is calculated as:
		Servings per day/3
Reduced-fat milk and milk products	≥three servings	Maximum score of 1 if intake is ≥three servings per day. If intake is <three servings, score is calculated as: Servings per day/3
Lean meat, poultry, seafood, eggs, nuts and seeds, and legumes	≥two servings	Maximum score of 1 if intake is ≥two servings per day. If intake is <two servings, score is calculated as: Servings per day/2

Total possible score for food-related recommendations: 10.

**Table 2:** Maternal characteristics and demographics of women with GDM included in the cohort.

Maternal Characteristic and Demographics	Total (*N* = 313)
Age in years ^1^	33.0 (29.0, 36.0)
20 to 24 years	13 (4.2)
25 to 29 years	66 (21.1)
30 to 34 years	114 (36.4)
35 to 39 years	91 (29.1)
40 years and over	29 (9.30)
Ethnicity	
European	146 (46.6)
Asian	98 (31.3)
Pacific People	34 (10.9)
Māori	31 (9.9)
Other	4 (1.3)
Socioeconomic deprivation	
1 to 2 (least deprived)	68 (21.7)
3 to 4	46 (14.7)
5 to 6	43 (13.7)
7 to 8	58 (18.5)
9 to 10 (most deprived)	98 (31.3)
Previous GDM	57 (18.2)
Primiparous	139 (44.4)
BMI	
Underweight	2 (0.6)
Normal	31 (9.9)
Overweight	103 (32.9)
Obese	177 (56.6)
Family history of diabetes	169 (54.0)
Smoking at trial entry	26 (8.3)
Gestational age at trial entry ^1^	31.4 (30.0, 32.4)

Numbers are *n* (%) or ^1^ median with interquartile range.

**Table 3:** Proportion of the 313 women with GDM who adhered to the guideline dietary recommendations and dietary adherence scores.

Dietary Recommendations	Fully Achieved ^1^	Median Score	25th Percentile	75th Percentile
Food-related recommendations				
Adherence to all food-related recommendations	0 (0.00)	6.17	5.34	7.00
Energy	253 (80.8)	1.00	1.00	1.00
Carbohydrate	264 (84.3)	1.00	1.00	1.00
Saturated fat	0 (0.00)	0.00	0.00	0.00
Vegetables	152 (48.6)	0.98	0.64	1.00
Fruits	142 (45.4)	0.93	0.52	1.00
Breads and cereals	39 (12.5)	0.44	0.29	0.71
Wholegrain breads and cereals	9 (2.9)	0.20	0.13	0.42
Milk and milk products	121 (38.7)	0.76	0.44	1.00
Reduced-fat milk and milk products	20 (6.4)	0.33	0.12	0.67
Lean meat, meat alternatives and eggs	107 (34.2)	0.79	0.57	1.00
Non-food-related recommendations				
Adherence to both non-food-related recommendations	78 (24.9)	*-*	*-*	*-*
Achieved recommended weight gain	88 (28.1)	-	-	-
Visited a dietitian	269 (85.9)	-	-	-

^1^ Numbers are *n* (%).

**Table 4:** Dietary adherence scores for energy, carbohydrate, and saturated fat intake, and association with maternal sociodemographic characteristics.

Maternal Characteristic	Total Dietary Adherence Score ^1^	Energy ^2^	Carbohydrate ^2^	Saturated Fat ^2^
Ethnicity	*p* = 0.992	***p* = 0.048**	*p* = 0.299	*p* = 0.351
European	6.20 (1.00)	0.96 (0.08) ^a^	0.97 (0.08)	0.06 (0.16)
Asian	6.12 (1.33)	0.94 (0.15) ^a^	0.96 (0.12)	0.06 (0.16)
Pacific People	6.21 (1.61)	0.98 (0.07) ^a^	0.98 (0.06)	0.11 (0.27)
Māori	6.18 (1.32)	0.94 (0.14) ^a,b^	0.97 (0.10)	0.04 (0.11)
Other	6.19 (1.61)	0.82 (0.26) ^b^	0.88 (0.24)	0.17 (0.24)
Maternal age in years	*p* = 0.139	*p* = 0.075	*p* = 0.359	*p* = 0.220
20 to 24 years	6.10 (1.38)	0.99 (0.05)	0.98 (0.07)	0.09 (0.23)
25 to 29 years	5.91 (1.09)	0.92 (0.16)	0.95 (0.12)	0.05 (0.14)
30 to 34 years	6.33 (1.22)	0.97 (0.10)	0.98 (0.09)	0.05 (0.14)
35 to 39 years	6.09 (1.28)	0.95 (0.11)	0.96 (0.10)	0.10 (0.21)
40 years and over	6.4 (1.15)	0.97 (0.10)	0.98 (0.09)	0.07 (0.19)
BMI	*p* = 0.212	***p* = ** **0.001**	***p* = ** **0.007**	***p* = ** **0.030**
Underweight	5.15 (1.70)	0.69 (0.11) ^a^	0.78 (0.17) ^a^	0.42 (0.18) ^a^
Normal	6.39 (1.09)	0.99 (0.03) ^b,d^	1.00 (0.00) ^b,d^	0.05 (0.15) ^b^
Overweight	6.30 (1.25)	0.94 (0.14) ^c,d^	0.96 (0.11) ^c,d^	0.07 (0.17) ^b^
Obese	6.08 (1.21)	0.96 (0.11) ^b,c,d^	0.97 (0.10) ^b,c,d^	0.06 (0.17) ^b^
Parity	*p* = 0.186	*p* = 0.128	*p* = 0.178	*p* = 0.486
Primiparous	6.07 (1.05)	0.94 (0.13)	0.96 (0.11)	0.07 (0.18)
Multiparous	6.26 (1.34)	0.96 (0.11)	0.97 (0.09)	0.06 (0.17)
Gestational age at trial entry ^3^	*p* = 0.993	*p* = 0.996	*p* = 0.896	*p* = 0.972
	R^2^ < 0.001	R^2^ < 0.001	R^2^ < 0.001	R^2^ < 0.001
Socioeconomic deprivation	*p* = 0.109	*p* = 0.588	*p* = 0.867	*p* = 0.200
1 to 2 (least deprived)	6.38 (1.05)	0.96 (0.10)	0.98 (0.08)	0.06 (0.17)
3 to 4	5.92 (0.87)	0.96 (0.08)	0.97 (0.10)	0.04 (0.15)
5 to 6	6.41 (1.40)	0.95 (0.13)	0.96 (0.11)	0.06 (0.16)
7 to 8	5.95 (1.18)	0.97 (0.10)	0.96 (0.10)	0.04 (0.14)
9 to 10 (most deprived)	6.18 (1.37)	0.94 (0.14)	0.96 (0.10)	0.10 (0.21)
Previous GDM	***p* = ** **0.021 ^4^**	***p* = ** **0.010**	***p* = ** **0.033**	***p* = ** **0.041**
Yes	6.50 (1.27)	0.99 (0.06)	0.99 (0.03)	0.02 (0.19)
No	6.09 (1.20)	0.95 (0.13)	0.96 (0.10)	0.07 (0.09)
Family history of diabetes	*p* = 0.100	*p* = 0.790	*p* = 0.742	*p* = 0.309
Yes	6.28 (1.26)	0.95 (0.12)	0.97 (0.10)	0.08 (0.19)
No	6.05 (1.16)	0.96 (0.12)	0.96 (0.10)	0.06 (0.15)
Smoking at trial entry	*p* = 0.103	*p* = 0.357	*p* = 0.618	*p* = 0.257
Yes	5.80 (1.19)	0.93 (0.12)	0.96 (0.09)	0.10 (0.23)
No	6.21 (1.22)	0.96 (0.12)	0.97 (0.10)	0.06 (0.17)
Achieved recommended weight gain	*p* = 0.941	*p* = 0.104	*p* = 0.211	***p* = ** **0.017**
Yes	6.17 (1.29)	0.94 (0.14)	0.96 (0.11)	0.10 (0.21)
No	6.18 (1.19)	0.96 (0.11)	0.97 (0.09)	0.05 (0.15)
Visited a dietitian	*p* = 0.715	*p* = 0.404	*p* = 0.676	*p* = 0.689
Yes	6.19 (1.25)	0.95 (0.12)	0.97 (0.10)	0.06 (0.17)
No	6.11 (1.04)	0.97 (0.09)	0.97 (0.08)	0.08 (0.19)

Numbers are *n* (%) or mean (SD); ^1^ from a possible minimum score of zero and a maximum score of 10; ^2^ from a possible minimum score of zero and a maximum score of one; ^3^ using linear regression; ^4^ bold *p*-values are significant at *p* < 0.05; ^a,b,c,d^ different letters indicate statistical differences (*p* < 0.05) on post-hoc comparison of dietary adherence scores in the same column between maternal characteristic groups.

**Table 5:** Maternal characteristics and demographics associated with dietary scores of the seven food groups.

Maternal Characteristic	Vegetable	Fruit	Milk and Milk Products	Lean Meat and Meat Alternatives	Bread and Cereal	Reduced-Fat Milk and Milk Products	Wholegrain Bread and Cereal
Median adherence scores ^1^	0.98 (0.64–1.00)	0.93 (0.52–1.00)	0.76 (0.44–1.00)	0.79 (0.44-1.00)	0.44 (0.29-0.71)	0.33 (0.12–0.67)	0.20 (0.13–0.42)
Ethnicity ^2^	*p* = 0.133	*p* = 0.871	***p* ≤ ** **0.001 ^4^**	*p* = 0.572	***p* = 0.001**	*p* = 0.506	*p* = 0.065
European	0.84 (0.20)	0.74 (0.31)	0.79 (0.24) ^a,d^	0.75 (0.23)	0.45 (0.23) ^a,d^	0.38 (0.32)	0.25 (0.18)
Asian	0.79 (0.26)	0.74 (0.30)	0.66 (0.29) ^b,d^	0.70 (0.35)	0.55 (0.30) ^b,c,d^	0.41 (0.34)	0.31 (0.27)
Pacific People	0.77 (0.32)	0.74 (0.30)	0.51 (0.37) ^c^	0.78 (0.29)	0.66 (0.33) ^b,c^	0.30 (0.35)	0.37 (0.34)
Māori	0.74 (0.30)	0.80 (0.30)	0.76 (0.26) ^a,b,d^	0.75 (0.29)	0.51 (0.33) ^a,b,d^	0.39 (0.36)	0.28 (0.28)
Other	0.92 (0.16)	0.79 (0.36)	0.75 (0.27) ^a,b,c,d^	0.81 (0.20)	0.35 (0.18) ^a,b,d^	0.48 (0.36)	0.23 (0.19)
Maternal age in years	*p* = 0.719	*p* = 0.929	*p* = 0.164	*p* = 0.157	*p* = 0.550	*p* = 0.771	***p* = 0.029**
20 to 24 years	0.74 (0.32)	0.78 (0.23)	0.56 (0.27)	0.76 (0.22)	0.53 (0.30)	0.34 (0.33)	0.33 (0.34) ^ab^
25 to 29 years	0.82 (0.26)	0.73 (0.30)	0.69 (0.28)	0.68 (0.35)	0.48 (0.25)	0.36 (0.34)	0.23 (0.18) ^a^
30 to 34 years	0.82 (0.24)	0.76 (0.30)	0.75 (0.29)	0.78 (0.27)	0.53 (0.29)	0.41 (0.34)	0.28 (0.24) ^a^
35 to 39 years	0.79 (0.25)	0.74 (0.33)	0.70 (0.29)	0.71 (0.27)	0.49 (0.28)	0.36 (0.33)	0.29 (0.25) ^a^
40 years and over	0.84 (0.23)	0.75 (0.28)	0.72 (0.28)	0.76 (0.21)	0.57 (0.30)	0.37 (0.31)	0.41 (0.31) ^b^
BMI	*p* = 0.324	*p* = 0.795	*p* = 0.861	*p* = 0.099	*p* = 0.456	*p* = 0.565	*p* = 0.129
Underweight	0.69 (0.26)	0.63 (0.43)	0.56 (0.62)	0.37 (0.02)	0.49 (0.50)	0.43 (0.57)	0.10 (0.09)
Normal	0.81 (0.27)	0.75 (0.27)	0.70 (0.27)	0.82 (0.23)	0.57 (0.26)	0.38 (0.32)	0.32 (0.22)
Overweight	0.84 (0.21)	0.77 (0.32)	0.72 (0.27)	0.74 (0.28)	0.53 (0.27)	0.42 (0.35)	0.32 (0.24)
Obese	0.79 (0.27)	0.74 (0.30)	0.72 (0.30)	0.73 (0.29)	0.49 (0.29)	0.36 (0.32)	0.26 (0.25)
Parity	*p* = 0.303	*p* = 0.848	*p* = 0.763	*p* = 0.148	***p* = 0.045**	*p* = 0.966	***p* = 0.018**
Primiparous	0.83 (0.24)	0.74 (0.30)	0.71 (0.27)	0.71 (0.29)	0.48 (0.26)	0.38 (0.33)	0.25 (0.20)
Multiparous	0.80 (0.25)	0.75 (0.31)	0.72 (0.31)	0.76 (0.27)	0.54 (0.30)	0.38 (0.34)	0.32 (0.27)
Gestational age at trial entry ^3^	*p* = 0.869	*p* = 0.339	*p* = 0.137	*p* = 0.859	*p* = 0.072	*p* = 0.862	*p* = 0.169
	R^2^ = 0.001	R^2^ = 0.003	R^2^ = 0.004	R^2^ < 0.001	R^2^ = 0.010	R^2^ < 0.001	R^2^ = 0.006
Socioeconomic deprivation	*p* = 0.798	*p* = 0.502	***p* = 0.033**	*p* = 0.262	*p* = 0.073	***p* = 0.032**	*p* = 0.280
1 to 2	0.83 (0.23)	0.78 (0.30)	0.79 (0.26) ^a,b^	0.75 (0.26)	0.52 (0.27)	0.43 (0.36) ^a,c,d^	0.29 (0.24)
3 to 4	0.78 (0.23)	0.79 (0.26)	0.71 (0.23) ^a,b,c,d^	0.69 (0.28)	0.43 (0.23)	0.29 (0.22) ^b,d^	0.24 (0.21)
5 to 6	0.83 (0.27)	0.76 (0.31)	0.77 (0.26) ^a,b,d^	0.80 (0.22)	0.51 (0.29)	0.47 (0.34) ^a,c^	0.28 (0.20)
7 to 8	0.82 (0.23)	0.71 (0.31)	0.65 (0.32) ^c,d^	0.76 (0.26)	0.48 (0.27)	0.32 (0.34) ^a,b,d^	0.25 (0.24)
9 to 10	0.80 (0.27)	0.72 (0.32)	0.68 (0.31) ^b,c,d^	0.70 (0.32)	0.57 (0.30)	0.39 (0.34) ^a,b,c,d^	0.32 (0.28)
Previous GDM	*p* = 0.790	*p* = 0.102	*p* = 0.768	*p* = 0.136	***p* = 0.010**	*p* = 0.651	***p* = 0.004**
Yes	0.82 (0.25)	0.80 (0.26)	0.72 (0.30)	0.79 (0.28)	0.60 (0.31)	0.40 (0.34)	0.37 (0.31)
No	0.81 (0.26)	0.73 (0.31)	0.71 (0.29)	0.73 (0.28)	0.49 (0.27)	0.38 (0.33)	0.27 (0.22)
Family history of diabetes	*p* = 0.181	*p* = 0.276	*p* = 0.819	*p* = 0.209	*p* = 0.687	*p* = 0.065	*p* = 0.971
Yes	0.83 (0.24)	0.77 (0.29)	0.72 (0.29)	0.75 (0.25)	0.51 (0.28)	0.42 (0.35)	0.29 (0.25)
No	0.79 (0.26)	0.73 (0.32)	0.71 (0.29)	0.71 (0.32)	0.50 (0.28)	0.35 (0.31)	0.29 (0.24)
Smoking at trial entry	*p* = 0.224	*p* = 0.679	*p* = 0.472	*p* = 0.093	*p* = 0.086	*p* = 0.615	***p* = 0.014**
Yes	0.75 (0.29)	0.72 (0.29)	0.68 (0.31)	0.65 (0.30)	0.42 (0.26)	0.41 (0.34)	0.17 (0.16)
No	0.81 (0.25)	0.75 (0.31)	0.72 (0.29)	0.74 (0.28)	0.52 (0.28)	0.38 (0.33)	0.30 (0.25)
Achieved recommended weight gain	*p* = 0.227	*p* = 0.312	*p* = 0.807	*p* = 0.177	*p* = 0.529	*p* = 0.306	*p* = 0.709
Yes	0.78 (0.25)	0.78 (0.28)	0.71 (0.30)	0.70 (0.30)	0.50 (0.27)	0.41 (0.34)	0.29 (0.26)
No	0.82 (0.25)	0.74 (0.31)	0.72 (0.29)	0.75 (0.27)	0.52 (0.28)	0.37 (0.33)	0.28 (0.24)
Visited a dietitian	*p* = 0.741	*p* = 0.688	*p* = 0.280	*p* = 0.389	***p* = 0.003**	*p* = 0.775	*p* = 0.171
Yes	0.81 (0.26)	0.74 (0.31)	0.71 (0.29)	0.73 (0.29)	0.53 (0.29)	0.38 (0.34)	0.29 (0.25)
No	0.80 (0.21)	0.76 (0.29)	0.76 (0.27)	0.77 (0.25)	0.40 (0.22)	0.37 (0.31)	0.24 (0.21)

^1^ Numbers are median (interquartile range); ^2^ remaining numbers are mean (SD) from a possible minimum score of zero and a maximum score of one; ^3^ using linear regression; ^4^ bold *p*-values are significant at *p* < 0.05; ^a,b,c,d^ different letters indicate statistical differences (*p* < 0.05) on post-hoc comparison of dietary adherence scores in the same column between maternal characteristic groups.

**Table 6:** Maternal characteristics and demographics associated with visitation to the dietitian and recommended weight gain.

Maternal Characteristics	Visited a Dietitian		Achieved Weight Gain	
	Adhered	Did Not Adhere	Adhered	Did Not Adhere
Proportion achieved adherence	269 (85.9)	44 (14.1)	88 (28.1)	225 (71.9)
Ethnicity	*p* = 0.156 ^1^		***p* = ** **0.037** ^1^	
European	121 (82.9)	25 (17.1)	44 (30.1)	102 (69.9)
Asian	86 (87.8)	12 (12.2)	32 (32.7)	66 (67.3)
Pacific People	33 (97.1)	1 (2.9)	9 (26.5)	25 (73.5)
Māori	26 (83.9)	5 (16.1)	2 (6.5)	29 (93.5)
Other	3 (75.0)	1 (25.0)	1 (25.0)	3 (75.0)
Maternal age in years	*p* = 0.663 ^1^		*p* = 0.182 ^1^	
20 to 24 years	12 (92.3)	1 (7.7)	4 (30.8)	9 (69.2)
25 to 29 years	59 (89.4)	7 (10.6)	15 (22.7)	51 (77.3)
30 to 34 years	99 (86.8)	15 (13.2)	26 (22.8)	88 (77.2)
35 to 39 years	74 (81.3)	17 (18.7)	33 (36.3)	58 (63.7)
40 years and over	25 (86.2)	4 (13.8)	10 (34.5)	19 (65.5)
BMI	*p* = 0.248 ^1^		*p* = 0.462 ^1^	
Underweight	2 (100.0)	0 (0.0)	1 (50.0)	1 (50.0)
Normal	30 (96.8)	1 (3.2)	9 (29.0)	22 (79.0)
Overweight	86 (83.5)	17 (16.5)	33 (32.0)	70 (68.0)
Obese	151 (85.3)	26 (14.7)	45 (25.4)	132 (74.6)
Parity	*p* = 0.860		*p* = 0.321	
Primiparous	120 (86.3)	19 (13.7)	43 (30.9)	96 (69.1)
Multiparous	149 (85.6)	25 (14.4)	45 (25.9)	129 (74.1)
Gestational age at trial entry ^2^	**OR (0.806) *p* = 0.011 ^3^**		OR (0.977) *p* = 0.723	
Socioeconomic deprivation	*p* = 0.349 ^1^		*p* = 0.083	
1 to 2	54 (79.4)	14 (20.6)	26 (38.2)	42 (61.8)
3 to 4	40 (87.0)	6 (13.0)	8 (17.4)	38 (82.6)
5 to 6	40 (93.0)	3 (7.0)	8 (18.6)	35 (81.4)
7 to 8	49 (84.5)	9 (15.5)	18 (31.0)	40 (69.0)
9 to 10	86 (87.8)	12 (12.2)	28 (28.6)	70 (71.4)
Previous GDM	*p* = 0.380		*p* = 0.515	
Yes	51 (89.5)	6 (10.5)	14 (24.6)	43 (75.4)
No	215 (85.0)	38 (15.0)	73 (28.9)	180 (71.1)
Family history of diabetes	*p* = 0.055		*p* = 0.288	
Yes	139 (82.2)	118 (90.1)	52 (30.8)	117 (69.2)
No	30 (17.8)	13 (9.9)	33 (25.2)	98 (74.8)
Smoking at trial entry	*p* = 0.554^1^		*p* = 0.544	
Yes	24 (92.3)	2 (7.7)	6 (23.1)	20 (76.9)
No	244 (85.3)	42 (14.7)	82 (28.1)	204 (71.3)

Numbers are *n* (%); ^1^ Fisher’s exact (<5 counts); ^2^ using logistic regression; ^3^ bold *p*-values are significant at *p* < 0.05.

## Data Availability

Published data are available to approved researchers under the data sharing arrangements provided by the Maternal and Perinatal Central Coordinating Research Hub (CCRH), based at the Liggins Institute, University of Auckland (https://wiki.auckland.ac.nz/researchhub, accessed on 31 May 2021). Metadata, along with instructions for data access, are available at the University of Auckland’s research data repository, Figshare (https://auckland.figshare.com, accessed on 31 May 2021). Data access requests are to be submitted to Data Access Committee via researchhub@auckland.ac.nz. Data will be shared with researchers who provide a methodologically sound proposal and have appropriate ethical and institutional approval. Researchers must sign and adhere to the Data Access Agreement that includes a commitment to using the data only for the specified proposal, to store data securely and to destroy or return the data after completion of the project. The CCRH reserves the right to charge a fee to cover the costs of making data available, if required.
